# Precision Gerontometry: Introduction, Fundamentals and Areas of Application

**DOI:** 10.3390/metabo16070463

**Published:** 2026-07-02

**Authors:** Petr G. Lokhov, Elena E. Balashova

**Affiliations:** Institute of Biomedical Chemistry, Pogodinskaya St. 10, Moscow 119121, Russia

**Keywords:** aging, metabolomics, biological clock, precision metabolic clock, mass spectrometry, healthspan

## Abstract

Aging is a major risk factor for numerous chronic diseases and a leading contributor to global mortality. Slowing the rate of aging would have revolutionary implications for health and longevity. A fundamental barrier to achieving this goal, however, is the difficulty of accurately measuring the effects of rejuvenating interventions. The development of precise gerontometric methods, therefore, is a priority for both science and preventive medicine. In this opinion article, the authors suggest the principles and discuss the implementation of precision gerontometry using recent advances in metabolomics. Although metabolomic approaches have limited accuracy in determining biological age, the described approach, which averages multiple metabolites from a large metabolomic signature of aging, circumvents this limitation. It allows for measurement of biological age change with an accuracy of approximately one month. Such precision gerontometry enables accelerated testing of candidate anti-aging interventions, helping to eliminate ineffective ones, speed the development of effective ones, and ultimately extend the duration of healthy human life, with profound social and humanitarian benefits.

## 1. Introduction

Aging, an inevitable natural process of body decline, is the primary factor in the development of many chronic diseases, which claim the lives of a significant portion of the population worldwide. Cardiovascular pathologies (heart attacks, strokes), neurodegenerative diseases (Alzheimer’s and Parkinson’s), cancer, type 2 diabetes, and osteoporosis are all inextricably linked to the accumulation of molecular and cellular damage characteristic of aging [1]. As the foundation for the development of major human diseases, aging is a key target for scientific research and preventive medicine.

Significantly slowing the rate of aging would have revolutionary consequences not only for individual health and longevity but also for society. Extending the period of healthy, active life would mean reduced healthcare costs, increased productivity, and, globally, significant humanitarian progress. People would be able to remain independent longer, contribute to society, and enjoy life for a long time without the severe ailments of age.

However, achieving this goal is hampered by a fundamental problem: *the inability to accurately measure the effect of potential anti-aging interventions in short-term studies*. Traditional clinical trials focused on specific diseases or mortality require years of research and huge sample sizes. Many existing biomarkers of aging (e.g., telomere length or DNA methylation) are insufficiently accurate and poorly reflect the response to short-term interventions [2]. As a result, *the effectiveness of most claimed anti-aging approaches is scientifically unproven* or seriously questionable. This creates fertile ground for speculation and unscrupulous marketing and hinders the development of truly effective approaches [3,4,5,6,7].

A breakthrough that could radically change the situation would be the development of a highly accurate method for measuring biological age. This is where modern analytical platforms come to the fore. The analysis of vast arrays of ‘omics’ data (genomics, epigenomics, transcriptomics, proteomics, metabolomics) makes it possible to identify complex patterns characteristic of biological age and the rate of aging [8]. Thus, the development of precise gerontometric instruments using omics approaches could become a catalyst for a revolution in anti-aging medicine. This would enable scientifically validated testing of hundreds of potential interventions, weeding out ineffective ones, accelerating the development of effective ones, and ultimately bringing us closer to the goal of extending the healthy lifespan of humans. This would also bring invaluable social and humanitarian benefits.

This article examines the problem of precisely measuring biological age and proposes, in the authors’ opinion, a mature solution based on the latest advances in metabolomics, which opens a new scientific and practical direction—precision gerontometry.

## 2. Problems of Biological Age Measurement by Biological Clocks

Accurately measuring biological age is a fundamental task in modern science and medicine. Accuracy is understood here as a correlation with chronological age, i.e., how well a model predicts chronological age in a healthy population (usually assessed using the coefficient of determination *R*^2^ or mean absolute error (MAE)). Let us consider the main types of existing biological clocks.

*Clinical/phenotypic clocks*, which are combinations of routine clinical parameters (blood pressure, cholesterol, glucose, liver/kidney function, pulmonary function tests, anthropometry) and/or functional tests (grip strength, gait speed, cognitive tests), have moderate accuracy (*R*^2^~0.5–0.7, MAE~5–8 years). The accuracy of these clocks is highly dependent on the choice of parameters, and sensitivity to biological age interference is estimated to be low. Changes in many clinical parameters may be nonspecific. This type of biological clock is characterized by relatively low accuracy in determining chronological age; it is susceptible to the influence of acute conditions; and it may not capture early molecular changes [9,10,11].

*Epigenetic clocks* operate by assessing the methylation of cytosine-phosphate-guanine (CpG) sites in DNA, which changes with age. This is the most advanced type of clock. The accuracy of chronological age determination is considered high (*R*^2^ > 0.95, MAE~2–4 years under optimal conditions for the best models, such as Horvath’s pan-tissue clock). These clocks also have good predictive power (e.g., GrimAge) and are sensitive to certain interventions [12,13,14,15].

*Transcriptomic clocks* operate based on gene expression patterns (mRNA) in tissues and blood cells and have moderate-to-high accuracy (*R*^2^~0.5–0.8, MAE~4–10 years), which depends on the tissue and technology used (RNA-seq is more accurate than microarrays) [16,17,18,19,20,21]. Disadvantages of these clocks include high technical variability, strong dependence on the cell/tissue type and current state (infection, stress), difficulty in standardization, and high cost (especially for RNA-seq).

*Proteomic clocks* are based on measuring circulating protein levels in plasma/serum. Their accuracy is moderately high (*R*^2^~0.6–0.9, MAE~2.5–6 years for complex models) [22,23,24,25,26]. Disadvantages include the high cost of complex analysis, technical difficulties, the influence of extraneous factors (diet, time of day), and high interindividual variability.

*Metabolomic clocks* use profiles of low-molecular-weight molecules (metabolites) in blood, urine, or other biofluids. Two primary approaches underpin metabolomic clock implementation: mass spectrometry (MS) and nuclear magnetic resonance (NMR) spectroscopy. MS offers broad and deep coverage, enabling the detection of hundreds of diverse, readily ionizable, low-concentration metabolites and providing profound biological pathway insights; however, many MS peaks remain unidentified. MS-based clocks show only moderate accuracy, typically achieving an *R*^2^ of ~0.45–0.64 and an MAE of ~4–8 years [27,28,29]. NMR-based approaches are characterized by high reproducibility—maintaining standardized outputs across batches—and strong clinical interpretability, as exemplified by Nightingale Health platforms [30,31,32,33,34]. The accuracy of these clocks is also considered moderate (*R*^2^~0.35–0.81, MAE~4–6 years). Metabolomic clocks are highly sensitive to ongoing exposures and are directly linked to metabolic health [35]. Disadvantages include enormous variability (affected by diet, time of day, and microbiome), difficulty in identifying metabolites and interpreting data. So far, they have lower accuracy and predictive power compared to the best epigenetic and proteomic clocks.

*Multiomic clocks* leverage multiple omic measurements simultaneously, enhancing predictive accuracy through the synergistic integration of data across distinct biological levels. The StackAge clock, which combines plasma proteins (Olink Explore platform) and metabolites (Nightingale Health platforms), leads this category in accuracy, achieving an *R*^2^ of 0.86 and an MAE of 2.4 years [36]. In the Human-Phenotype Project cohort, the integration of transcriptomics, metabolomics, and microbiome data yielded an *R*^2^ of 0.79 and an MAE of 4.0 years [37]. The combination of urinary metabolites (MS) and gut microbiota composition (16 S rRNA) resulted in an *R*^2^ of 0.50 and an MAE of 4.9 years [38]. A joint regression model incorporating the blood metabolome and an epigenetic clock (DNA methylation) demonstrated an *R*^2^ of 0.74 and an MAE of 3.7 years [39]. The multi-organ molecular clock, which integrates macrostructural imaging data (MRI of the brain, heart, liver, and kidneys) with molecular profiles (plasma proteins and metabolites), showed an *R*^2^ of 0.60 and an MAE of 5.0 years [40]. Finally, the multimodal clock of human aging, which utilizes clinical, physiological, and multilevel molecular data (DNA methylation, transcriptomics, and proteomics), exhibits an *R*^2^ of 0.90 and an MAE of 3.9 years [41].

**Figure 1:**
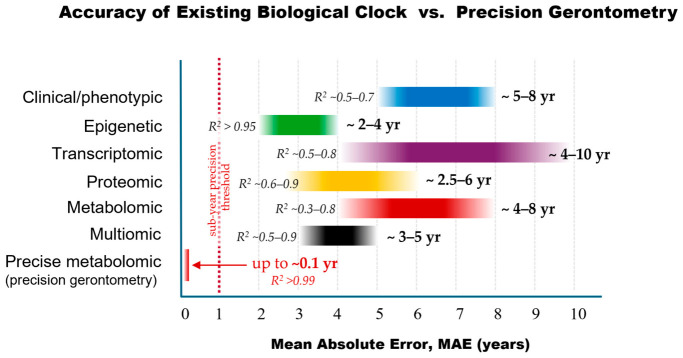
Mean absolute error (MAE) for different biological clocks. Reported MAE ranges for the six major clock families are compared with the precise metabolomic approach proposed in the present work, whose error is approximately one month (36 days; ≈0.1 year). The MAE ranges are a rough summary of the MAE values presented in the studies cited in Section 2. They illustrate the conceptual difference between precision gerontometry and the six main types of biological clocks. The dashed red line marks the sub-year precision threshold below which existing clocks cannot operate.

Figure 1 summarizes the reported accuracy of these six clock families on a common scale, visualizing the accuracy issue that motivated this work. As we can see, the accuracy of biological age measurement depends heavily on the chosen method. However, none of the chosen options is precise, as the MAE is measured in years, which explains the significant limitations of their practical application.

If the accuracy of a method for measuring biological age in response to any influence on the body is measured in years, then such low resolution forces an experimenter to choose between two impractical options. Option 1—use an exceptionally strong intervention that changes biological age by many years in a short period—is unrealistic, because no such intervention has been described to date. Option 2—run the experiment for many years (or decades)—is undermined by the inevitable accumulation of confounding lifestyle, dietary and environmental influences during such a long observation window. In measurement science, this corresponds to the well-known drift problem, where extended observation accumulates both Type A (random) and Type B (systematic) uncertainty components) [42,43,44]. Thus, *the problem of searching for anti-aging remedies lies in the lack of a method for measuring biological age with significantly greater accuracy than existing methods*. Therefore, the development and validation of a truly accurate biological clock is a critical step in the science of aging. Given the predominant need to measure the aging of an organism, rather than measuring biological age at its inception and maturation, we are essentially talking about precision gerontometry.

## 3. Fundamentals and Features of Precision Gerontometry

This section is organized into two layers. Section 3.1 first sets out the three theoretical Bases that make precision gerontometry possible. Section 3.2 then translates these Bases into seven operational Principles for routine measurement, and the subsequent subsections (3.3–3.9) describe how Principles are implemented in practice.

### 3.1. The Fundamentals for Precision in Gerontometry

#### 3.1.1. Basis #1—Aging Is a Deeply Systemic Process

It is a well-known fact that aging is a deeply systemic process affecting the entire body (all organs, tissues, cells, anabolic and catabolic processes, signaling pathways, etc.). Therefore, the metabolome, which is considered the molecular phenotype of the organism, is also deeply affected by aging. This is confirmed by the fact that the MetaboAge database (www.metaboage.info, accessed on 1 January 2026) [45] was created to catalog aging-associated metabolites, which is not typical for individual diseases. Moreover, a recent study has shown that age-related changes simultaneously affect several specific metabolic pathways that are also statistically significantly associated with disease. This allowed the authors of the study to identify an aging- and health-related *metapathway*, a term chosen to emphasize that aging affects a specific and significant part of the entire human metabolic network [46]. Such wide reflection of aging in the metabolome is the reason for the existence of the next, second basis of precision gerontometry.

#### 3.1.2. Basis #2—Large Signature of Deeply Systemic Process Gives Stable Precise Signal

In metabolomics, the principle is that measuring multiple metabolites (a metabolite panel or signature) yields a more reliable and accurate result than measuring a single metabolic marker. This effect is achieved through mutual compensation of random and other undesired fluctuations and the elimination of technical or biological interference [47,48,49,50,51,52].

Therefore, from the abundance of metabolites from the age-related part of the metabolome, one signature, a *large aging signature*, can be compiled. The main advantage resulting from its large size is the signal stability that the large signature provides. As an example, in a recent study, precision gerontometry was proposed as a control for metabolomics-guided nutrition. In that study, such a large signature included the intensity values of 3245 mass spectrometry peaks for men and 1838 peaks for women [53].

Given that the precision of biological age measurement depends on the number of *measured* metabolites (not to be confused with the number of *identified* metabolites), blood metabolome fingerprinting is the method of choice for determining biological age. In this context, *a fingerprint is a multivariate characteristic formed by the intensities of mass spectrometric peaks of metabolites* of a particular biosample. The selection of blood as the sample is obvious. Blood is readily available for analysis; blood collection and transportation are widely used in practice; metabolites are transported through the blood; cells receive substances from the blood and remove waste products into the blood; and substances with signaling properties also act through the blood. Thus, the blood metabolome, with minor assumptions, can be considered representative of the metabolome of the entire organism.

**Figure 2:**
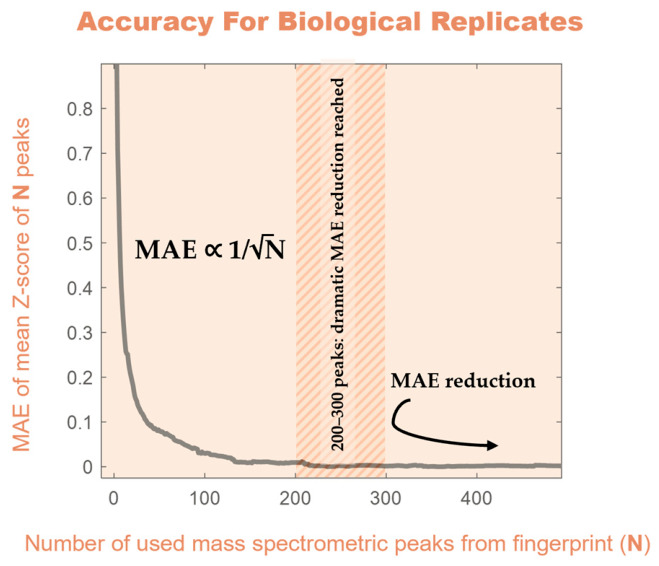
Reduction in the mean absolute error (MAE) depending on the number of the used mass spectrometric peaks from the metabolomic fingerprint. The plot was drawn using previously published mass spectrometric data for biological replicates of blood plasma samples [53]. The Z-score represents the intensity of mass spectrometry peaks on a dimensionless scale. The shaded region indicates the range of 200–300 peaks, at which a dramatic reduction in MAE is reached.

The absence of metabolite identification (only detection) in metabolome fingerprinting allows involving thousands of blood metabolites, including those at low concentrations, which is important for detecting large signatures. Figure 2 shows that using approximately 200–300 mass spectrometric peaks of a metabolomic fingerprint results in a dramatic reduction in the mean absolute error (MAE) for biological replicates characterizing biological reproducibility. Moreover, the data for biological replicates are very closely described by the equation MAE = 1/√N, where N is the number of peaks used from the fingerprint. This occurs by leveling out the fluctuations of individual metabolites through averaging and using the mean value as a stable signal for precision measurements. Since precision gerontometry requires the use of only metabolites correlated with age, fingerprinting can enable the detection of hundreds of such metabolites that form a large aging signature.

#### 3.1.3. Basis #3—Measure Not Biological Age but Its Change

There is a fundamental problem with the precision of biological clocks. Chronological age is used to construct and characterize their precision. However, biological age is not equivalent to chronological age (that is why a biological clock is created). Therefore, to create a precise biological clock, an exact biological age should be known. Yet, this would require using the same kind of precise clock to determine that age, which is logically impossible.

To achieve precision, it is necessary to rethink the principle of measuring biological age. Precision gerontometry is only possible when parameters of the same individual are measured (i.e., in a prospective study). First, the rate of natural aging (the change in biological age over a certain period) is measured. Then, after exposure to the tested intervention, the rate of aging is measured again in the same individual. If a statistically significant difference between the two measurements is detected, a conclusion is drawn about the geroprotective or gerotoxic properties of the intervention. For greater clarity, it can be said that if the described approach for precision gerontometry is used to estimate the absolute value of biological age, then this will be a metabolomic clock that does not demonstrate high precision.

**Figure 3:**
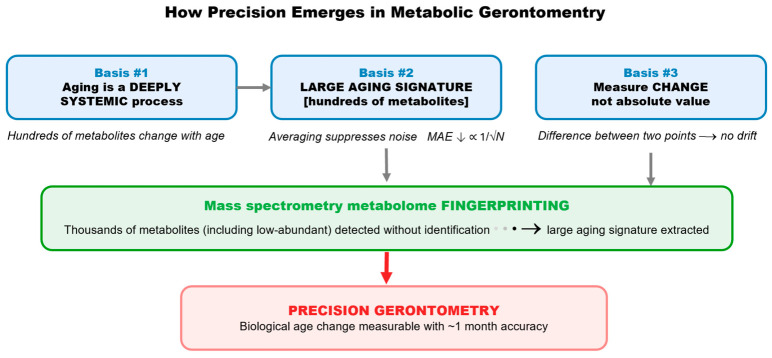
Schematic explanation of the precision appearance in measuring biological age using metabolomics. The three Bases (aging is a deeply systemic process; a large aging signature consisting of hundreds of metabolites; measuring change rather than absolute age) jointly enable mass spectrometry metabolome fingerprinting to yield precise gerontometric measurements with approximately one-month precision.

Figure 3 summarizes the above fundamentals and demonstrates in one general diagram the emergence of precision in measuring biological age using metabolomics.

### 3.2. Principles of Precision Gerontometric Measurements

Starting from the above-mentioned fundamentals, we attempted to formulate the principles of precision gerontometric measurements:

1. It is necessary to measure not biological age, but only its change in the same person.
2. All mass spectrometric measurements are performed on the same instrument and using the same protocol, to minimize technical and biological variability.
3. Measuring biological age must involve the age-related dynamics of the metabolome, represented by an age-related metabolomic curve.
4. The use of specially selected volunteers (gerovolunteers) is required (for the definition and description of gerovolunteers, see Section 4).
5. Signal stability for precision measurement is achieved by averaging the metabolomic fingerprint data forming the large aging signature.
6. Metabolomic fingerprint processing must be performed with low alignment error rates.
7. Minimum detectable change (MDC) in biological age is 1 month or less.

As can be seen, the principles of precision gerontometric measurements, in addition to the fundamentals, contain several methodological principles, which we explain below.

### 3.3. Age-Related Dynamics of Blood Metabolome

In a previous study, to identify age-related dynamics of the metabolome, the integral metabolomic curve was built. For this, blood plasma metabolomic fingerprints of individuals of different ages were obtained and processed as follows [53]:

1. Selecting age-related (correlated) mass spectrometric peaks (large aging signature formation).
2. Smoothing (removing noise and unnecessary individual variability) of the mass spectrometric peak intensities by obtaining a moving average over age with a 7-year period.
3. Converting the mass spectrometric peak intensities into dimensionless Z-scores (for further joint processing).
4. Obtaining the mean Z-score for each fingerprint ($\bar{Z}$-score—an integral metabolome value) and plotting their dependence on age (plotting an age-related metabolomic curve).

**Figure 4:**
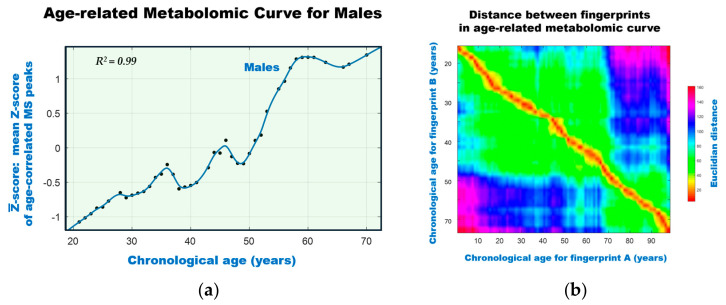
Age-related dynamics of human blood metabolome. (**a**) Age-related metabolomic $\bar{Z}$-score curve. $\bar{Z}$-score is the mean intensity of age-correlated mass spectrometric peaks, represented by the Z-score. *R*^2^ is the coefficient of determination for curve fitting. To construct the curve, samples from 96 males aged 18 to 74 years were used. Panel (**b**) shows the Euclidean distances between the metabolomic fingerprints used to construct the age-related metabolomic curve in (**a**). This panel shows that although the $\bar{Z}$-score curve has waves and the same $\bar{Z}$-scores can correspond to different ages, the metabolomic fingerprints are specific at each age point. Adapted from [53].

Figure 4a below shows an example of an age-related metabolomic curve obtained for males using blood plasma samples. Similar curves obtained for women and for both genders using dried blood spot samples can be seen in Ref. [53].

The figure visualizes the age-related properties of the blood plasma metabolome. Wave-like dynamics are observed up to age 50 for men (and 47 for women [53]), followed by a linear change, leading to metabolome fluctuations at a new level. Notably, the nonlinear dynamics of the metabolome with age and the timing of the waves are consistent with recently published data [54,55]. However, despite the wave-like shape of the integral curve reflecting the dynamics of the metabolome, Figure 4b confirms that the metabolomic fingerprints that form the curve are specific to each age point. This means that the large aging signature is specific for every age and can be used to measure biological age despite the wave-like fluctuations of the integral metabolome value.

The construction of the age-related metabolomic curve not only visualizes the dynamics of blood metabolome aging and prepares the mass spectrometric setup for gerontometric measurements, but also, as indicated in Section 3.5, is necessary for determining biological age change.

The quality of an age-related metabolomic curve depends on the source data—specifically, the number of subjects used and the accuracy with which their chronological age is recorded. Existing evidence suggests that two subjects per year of age are sufficient. By averaging the data used to construct the metabolomic curve (both for subjects of the same age and for age groups; see point 2 on smoothing), it was possible to remove noise. This led to achieving a minimum detectable change (MDC) in biological age of one month, and building a precise metabolic clock [53]. Using a larger number of individuals with more accurate chronological age information (for example, specifying not only the year but also the month of birth) could allow the construction of an even higher-quality age-related metabolomic curve.

### 3.4. Mass Spectrometry in Precision Gerontometry

Since the precision of measuring changes in biological age is ensured by measuring a significant number of metabolites, high-resolution mass spectrometry must be used for this purpose. Such mass spectrometry can be implemented using, for example, high-resolution time-of-flight mass spectrometers (HR-TOF; resolving power (FWHM, Full Width at Half Maximum) ~20,000–100,000). Other options include mass spectrometers with high accuracy and resolution, such as Fourier transform ion cyclotron resonance (FT-ICR; FWHM~0.5–1 × 10^6^), Orbitrap (FWHM~7500–500,000), or hybrid quadrupole-time-of-flight (Q-TOF; FWHM~10,000–100,000) mass spectrometers. Mass spectrometers with low resolving power are less suitable or may be entirely unsuitable (e.g., quadrupole or ion trap mass spectrometers; FWHM~500–10,000). The question of using chromatography for preliminary separation of metabolites remains open. Although chromatography increases the dynamic range of detection, as an additional step, it introduces extra variability into the data, worsens multivariable data reproducibility, and may complicate the routine application of gerontometry [56]. Apparently, until data on gerontometry using chromatography appears, direct high-resolution mass spectrometry is currently the most justified.

### 3.5. Features of Mass Spectra Processing in Precision Gerontometry

If there is a significant number of peaks in each high-resolution mass spectrum, it is necessary to establish their correspondence with reference peaks, which is achieved by mass peak alignment. In widespread metabolomics approaches (for example, in case–control and targeted metabolomic studies), alignment errors are usually not critical, because the peaks of interest are few and their alignment can be adjusted manually if necessary. However, when high-resolution mass spectrometry is used to obtain a large signature involving thousands of detected peaks (as required by precision gerontometry), alignment errors become a quantitatively significant source of bias. For example, a 1% error in aligning a mass spectrum consisting of 10,000 peaks results in 200 errors. This corresponds to 100 detected peaks where there should be blanks, and blanks in 100 places where there should be detected peaks. Moreover, often an alignment error does not result in assigning a peak to an empty position, but in erroneously assigning its value to another peak, which also increases the number of errors.

From a gerontometry perspective, this is a significant distortion of the raw data of large signatures. Thus, mass spectrometry practice, in which measurements are performed using protocols suitable for a wide range of tasks and sample types, and using standard, widely used alignment software, is not suitable for precision gerontometry. In our practice, the required low level of alignment errors was achieved by using mass spectrometers of the same type and brand with the same direct-infusion mass spectrometry protocol for 15 years. During this long period, we have continuously refined our specialized in-house software for mass spectra alignment, making it possible to virtually eliminate errors.

### 3.6. Biological Age Change Measurement and Minimum Detectable Change (MDC)

**Figure 5:**
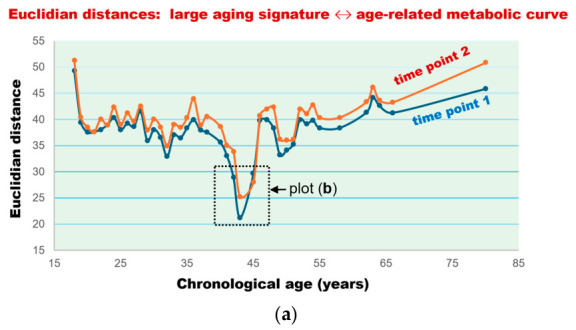

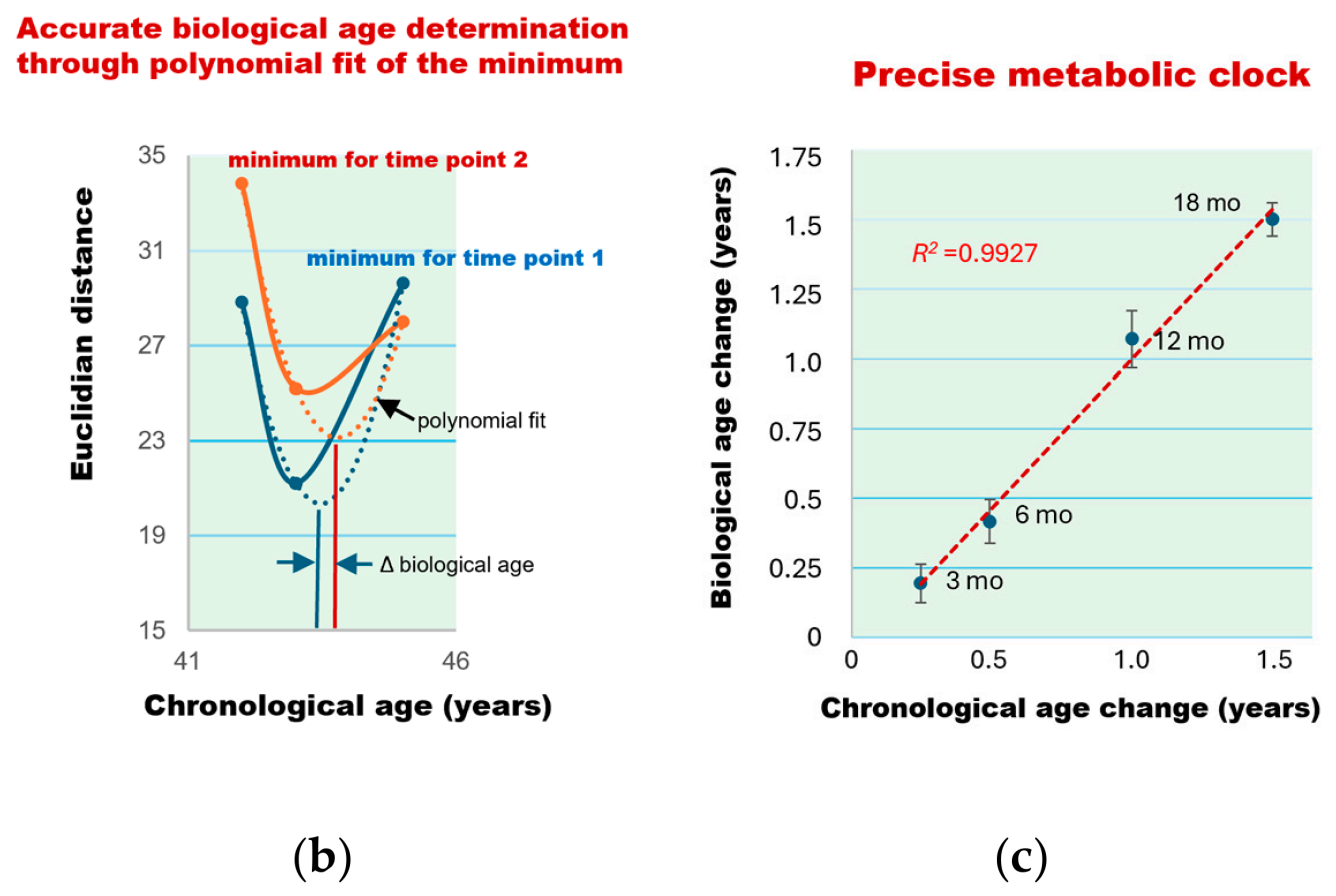
An example of biological age change calculation as the difference in biological age between two time points (red and blue colors). (**a**) The distances (Euclidean distance) between an individual’s metabolic fingerprint and each point on the age-related metabolomic curve. (**b**) The three points forming the minimum of the curves presented in panel ‘a’. The minimum value of the polynomial extrapolation of these points more accurately indicates biological age at two time points, the difference between which is suitable for precision gerontometry. (**c**) Comparison of biological age change measurements using the presented approach with chronological age change on a sub-annual scale (proof of concept of a precise metabolomic clock). *R*^2^—coefficient of determination for line extrapolation. Adapted from [53].

Measuring the change in an individual’s biological age using an age-related metabolomic curve is proposed in the most simplified way. The distance (Euclidean distance) from an individual’s large aging signature to each point on the curve is measured. Where the similarity is greatest, the distance is minimal, indicating the biological age of the individual. Considering that the age-related curve in the given example is constructed with a one-year scale division, extrapolation from three points that form a minimum can be used to accurately determine biological age. The technical variability of this approach is quite low, and the MAD is only 12 days (CI 95% 8.9–15.3) [53]. An example of such a distance measurement is shown in Figure 5a. Figure 5b shows an example of such an extrapolation for accurately determining biological age and measuring the change in biological age as the difference in its values between two time points.

Figure 5c demonstrates the validation of precise biological age measurements as they are linearly related to changes in chronological age (the coefficient of determination *R*^2^ for linear extrapolation was 0.99). This can be considered a proof of concept of precision gerontometry, since a sub-annual scale was used.

Of course, precision gerontometry is not limited to the presented bioinformatics processing of fingerprints for measuring biological age change, and researchers can use other approaches. However, the use of distance calculations between a gerovolunteer’s large aging signature and all points on the age-related metabolomic curve to obtain a signal indicating biological age is not accidental. When using a low-quality fingerprint (for example, the gerovolunteer’s metabolome is significantly altered by external or internal factors or due to problems with the mass spectrometry quality), the signal weakens or disappears. However, no shift in the signal along the age scale or the appearance of a false signal is observed. This is because any distortions introduced into the fingerprint are not specific to any particular fragment of the age-related metabolomic curve. So the signal (i.e., the minimum of the curve in Figure 5a) is simply smoothed out. In this way, it is possible to have both an error-resistant signal and to evaluate its quality (i.e., to have quality control for precise age measurement). When using other bioinformatics processing, researchers should strive to use algorithms demonstrating similar properties to maintain quality control and measurement precision.

### 3.7. General Workflow for Precision Gerontometry

Considering that metabolite measurement in a sample can be conducted in different ways, the approach we propose can be considered as a possible, but not limiting, option for implementing gerontometric measurements.

Before commencing gerontometric measurements, an age-related metabolomic curve should be constructed using direct high-resolution mass spectrometry of plasma samples or dried blood spots obtained from volunteers of different ages and both genders. This inclusion of both genders is only necessary if gerontometric measurements are planned for both genders. Examples of protocols can be found in a previously published article [53]. The selection of volunteers for this purpose follows the general rules applied in scientific research. While there are no strict requirements for volunteers participating in the construction of the age-related metabolomic curve, since this is a population characteristic. However, subsequent gerontometric measurements require the participation of gerovolunteers (see Section 4).

**Figure 6:**
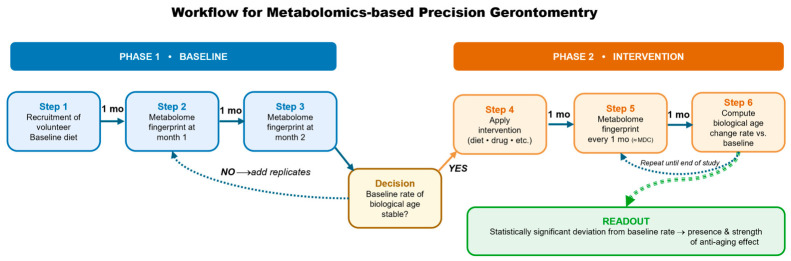
General workflow for metabolomics-based precision gerontometry. The protocol begins with adherence of the gerovolunteer to a baseline diet (Phase 1—BASELINE). After one and two months, metabolomic fingerprints are obtained, and the baseline rate of biological-age change is assessed. If results are unclear, additional biological replicates and time intervals are added. Once the metabolic fingerprint and its change rate are confirmed to be normal, the studied intervention is applied (Phase 2—INTERVENTION), and biological age is measured at one-month intervals. A statistically significant deviation from the baseline rate of biological age change is used to detect the presence and strength of the anti-aging effect of the intervention. The one-month measurement interval is determined by the minimum detectable change (MDC) in biological age, calculated from experimental data [53].

The gerontometry protocol begins with gerovolunteers’ adherence to a baseline diet (for example, the Mediterranean diet [53]), followed by metabolome fingerprints obtained after one and two months for assessing the baseline for biological age change rate. If the results are unclear, additional biological replicates and time intervals are used. After confirming a clear signal for measuring biological age and the normal aging rate, the intervention being studied is applied, and biological age is measured at monthly intervals. The monthly measurement intervals are determined by the minimum detectable change (MDC) in biological age change, which was calculated based on experimental data [53]. A statistically significant deviation from the baseline rate of biological age change is used to determine the presence and strength of the anti-aging effect of the studied intervention (Figure 6).

### 3.8. Biological Confounders

Precise gerontometric measurements must consider biological factors influencing the composition of the blood metabolome. The study conducted by Bar and coauthors in 2020 has shown that genetics, the gut microbiome, clinical parameters, diet, lifestyle, and anthropometric measurements collectively explain more than 76% of the variation in the blood metabolome [57]. Among these, diet and the microbiome exert the strongest influence, accounting for more than 50% of the observed variation. In the context of gerontometry, which deals with a prospective study on the same person, the influence of genetics can be neglected. The genome does not change in the same individual over the entire period of gerontometric measurement. Similarly, lifestyle and anthropometric factors are expected to remain stable during the gerontometric study, which is ensured by the participation of compliant gerovolunteers.

Since diet and the microbiome (which is largely determined by diet) are the main determinants of the blood metabolome, dietary changes are unacceptable during gerontometric studies. Furthermore, blood sampling should be performed on an empty stomach, as food intake, although not significantly (close to normal biological variability [53]), influences the measured change in biological age.

Although diet, the most significant determinant of blood metabolome variability, has a limited effect on the large aging signature, it is expected that less pronounced factors will be able to be ignored in gerontometric measurements. These factors include all other factors influencing the metabolome, including medication use, physical activity, circadian rhythms, acute illnesses, and environmental exposures. However, this is still only a hypothesis that requires further confirmation.

### 3.9. Application of Dried Blood Spot Samples

The potentially widespread application of precision gerontometry makes it appropriate to evaluate its compatibility with dried blood spot (DBS) samples. DBS samples are collected at home, stored and transported to the laboratory in a dried form at room temperature. An assessment of biological variability of biological age determination by metabolic fingerprinting yielded an MDC of 27.7 days for using DBS [53], which corresponds to the precision of the measurement. The MDC is the smallest change in a measurable value that reliably exceeds random measurement error or system “noise.” Therefore, the anti-aging effect can be recorded if it consists of a change in biological age of one month or more. In this situation, it is advisable to monitor biological age change using DBS no more than once a month.

### 3.10. Implementation and Control of Precise Gerontometric Measurements

Omics technologies are powerful but difficult to translate into clinical tests due to multistep complexity, expert requirements, and standardization issues. Consequently, the few omics tests used clinically are laboratory-developed tests (LDTs) limited to single laboratories. This is because complex multivariate readouts are difficult to reproduce or verify by regulators and vary even within the same laboratory, making standardization and inter-lab transfer challenging [58,59,60]. These omics tests remain local and transient, creating an existential gap between omics’ prominence in biomedical research and their negligible clinical application.

Precision gerontometry, a successful metabolomic translation, overcomes this. A true omics test, it delivers one clear parameter—biological age change. If a laboratory claims the ability to measure biological age with an accuracy of one month, one need only supply four or five blinded blood samples drawn from the same individual at one-month intervals. Should the gerontometric measurement correctly reconstruct the chronological order of sample collection, the laboratory qualifies as a gerontometric facility. The probability of randomly arranging four samples in the correct order is merely *p* = 0.04; for five samples, it drops to *p* = 0.008. This verification procedure is suitable for external control by the regulator, customer control and routine internal control. Moreover, successfully passing such a test constitutes indisputable proof of concept for precision gerontometry.

## 4. Gerovolunteers

**Figure 7:**
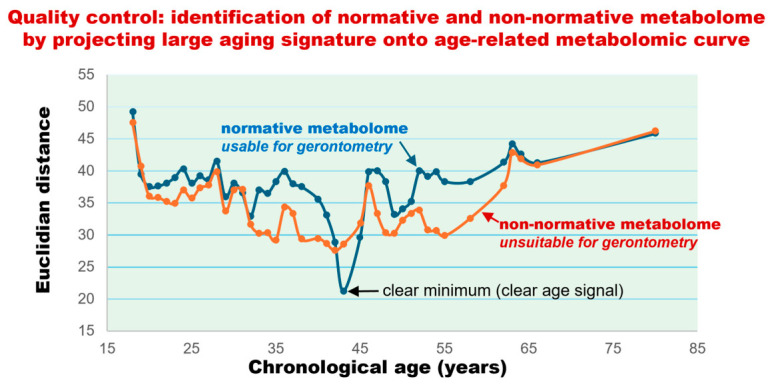
The Euclidean distances between the large aging signature of normative and non-normative individuals’ metabolomes and each point on the age-related metabolomic curve. Metabolome data from men of the same age (43 years) were used. The normative metabolome provides a clear signal (a distinct minimum) from which biological age can be calculated. The non-normative metabolome lacks a clearly defined signal and is not suitable for gerontometry. Data from the published study were used [53].

As anticipated in Section 3.2 (Principle #4), routine application of precision gerontometry requires a specially selected pool of volunteers (gerovolunteers). Their stable physiology preserves the signal-to-noise advantage of the method. These individuals are men and women with a stable normative blood (plasma) metabolome that also changes normatively over time (Figure 7). Gerovolunteers follow a baseline diet and have no acute or chronic diseases affecting the blood metabolome. The Mediterranean diet is proposed as the baseline diet, as it has previously been shown to be the most balanced and neutral in relation to the aging-related part of the human metabolome [53]. Throughout the entire gerontometric study period, gerovolunteers must adhere to the same specific, unchanging diet and lead a stable lifestyle.

Before conducting gerontometric measurements, gerovolunteers undergo blood metabolome analysis at a specific time interval to confirm the typicality of their metabolic fingerprints and the normal rate of biological age change. The deviation from this rate is used to assess the geroprotective properties of the intervention that caused this deviation.

## 5. Precision Gerontometry vs. Pace-of-Aging Measurements

Pace-of-aging metrics measure how fast your body’s systems decline relative to chronological time: a value of 1.0 means one biological year per calendar year. Unlike biological age clocks, pace of aging is not expressed as an age; therefore, MAE is reported in ‘pace units per year’ and not compared with MAE of biological clocks (Figure 1). DunedinPACE, the leading pace-of-aging method based on DNA methylation [15], achieves a MAE of just hundredths of a unit, making it highly accurate for tracking anti-aging interventions within months. Precision gerontometry is comparable but still slightly less accurate. However, DunedinPACE uses blood cells and reflects aging of particular cells, not whole-body aging. Because different cells age at different rates, the method is applied to various biospecimens with different results [61]. If the anti-aging effect affects the body’s cells differently or does not affect the cells used in the measurement, then the epigenetic approach will give a false result. In contrast, gerontometry analyzes whole blood or plasma, which serve as systemic collectors and treat the metabolome as a molecular phenotype of the entire organism. Moreover, there is no fundamental obstacle to extending gerontometry to the metabolome of specific cells; this could yield an even smaller MAE and merit direct comparison with DunedinPACE.

## 6. Application of Precision Gerontometry

Precision gerontometry, i.e., precise measuring of biological age change in response to interventions, in the authors’ opinion, has enormous translational potential. Its possible applications can be broadly categorized into the areas presented in Table 1.

**Figure 8:**
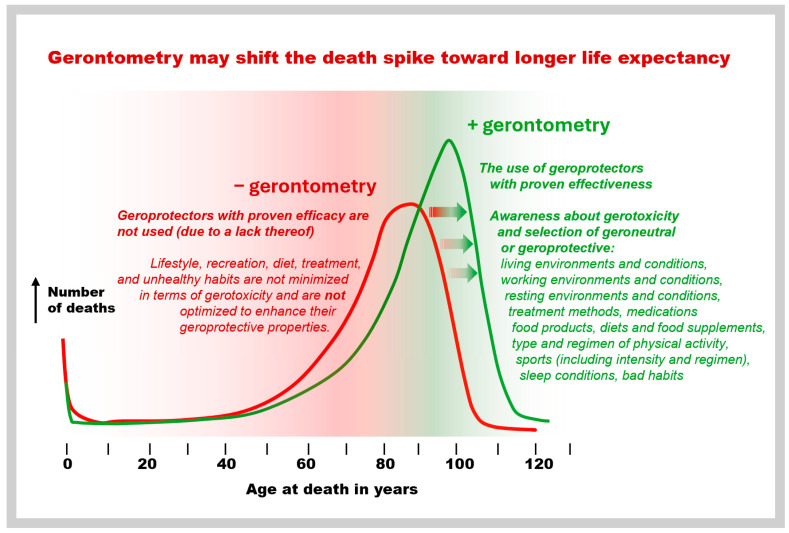
A possible change in human life expectancy and living conditions with the widespread use of precision gerontometry.

As the table shows, gerontometry potentially has a wide range of applications—and this is no coincidence. Years lived are a natural, universal, and currently the only measurement of life. In principle, all factors, both external and internal, can be divided into geroprotective and gerotoxic; the only difference lies in the strength and duration of their influence. With a tool to measure these factors, a person can make life expectancy manageable. Instead of assumptions and speculation, we can precisely classify influences as geroprotective or gerotoxic and measure their effects. This could mark a turning point in life expectancy (Figure 8) and hold enormous potential for improving economic performance. Benefits include lower healthcare costs, higher productivity and tax revenues, optimized pension systems, and new economic sectors. Developing precision gerontometry will allow us to actively join the global race to slow aging, using it as a key tool for humanitarian and economic success.

## 7. Final Notes

It seems appropriate to say a few words about how gerontometry differs from gerontology. It is related to gerontology in the same way that, for example, biometrics is related to biology, or psychometrics is related to psychology. The key difference is hidden in the name itself: Gerontology (from the Greek *geron*—old man, and -*logy* from *logos*—the study of) is the comprehensive science of aging. Gerontometry (*-metry* meaning “the process of measuring”, from the Greek *metron*—measure) is an applied discipline focused exclusively on measuring aging. However, to avoid confusion with the imprecise methods of measuring aging that exist in gerontology, and to emphasize the key difference related to the accuracy of measurements, it seems appropriate to use the complex term *precision gerontometry*.

Another note concerns the application of precision gerontometry to animal models. This is especially relevant in the case of the search for new geroprotectors, since human trials are traditionally preceded by tests in animals. According to previous research, profound age-related changes in the metabolome are virtually identical across different aging models, from nematodes to humans [62]. Thus, the fundamentals for obtaining precision presented in Figure 3 are complementary across this wide range of animal models, and the findings from them are applicable to humans. This makes precision gerontometry a full-featured research tool for studying aging and developing anti-aging treatments.

One of the strengths of precision gerontometry compared to epigenetic pace-of-aging measurements is that it utilizes the blood plasma metabolome as an integral molecular phenotype, which is unavailable with epigenetic clocks. However, this can also be viewed as a limitation, as epigenetics clearly shows that different cells and organs have different rates of aging. Therefore, if the aging of individual cell types does not affect the blood metabolome but is of interest, then the development of gerontometric cell measurements will be relevant.

Although the emergence of precision gerontometry is a largely logical and mature direction of metabolomics, it is in its infancy. Therefore, the demonstrated precision parameters, limiting factors, and the effectiveness of its application in various fields, presented in Table 1, require further extensive testing. We hope that the promising accuracy and potential wide application will facilitate the implementation and dissemination of this direction.

## 8. Conclusions

The development of precision gerontometric instruments is a pressing challenge in modern science and preventive medicine. The most promising approaches are “omic” methods, which currently have significant limitations in the accuracy of biological age determination. However, the use of metabolomic measurements, based on knowledge of metabolome dynamics with age and expertise in processing multivariable metabolic parameters, allows for a dramatic increase in measurement accuracy. The development of a precise gerontometer, therefore, promises revolutionary changes in anti-aging medicine and leads to the emergence of a new scientific field—precision gerontometry. This will enable scientifically validated testing of hundreds of potential interventions, weeding out ineffective ones, accelerating the development of effective ones, and, ultimately, bringing us closer to the goal of extending the healthy lifespan of humans, bringing invaluable social and humanitarian benefits.

## Figures and Tables

**Table 1:** Areas of possible application of precision gerontometry.

Development and Clinical Trials of True Geroprotectors
Search for new medicinal compounds targeting fundamental mechanisms of aging (senolytics, mTOR inhibitors, autophagy activators, mitochondrial drugs, etc.). Accelerating the development of effective drugs that slow aging and prevent many age-related diseases. Reducing the time and cost of clinical trials by using biological age as a surrogate endpoint. Prevention/treatment of the leading causes of mortality and disability. Rapid screening of ineffective or toxic candidates, saving money on screening unpromising developments.
Repurposing existing drugs
Assessing the potential geroprotective properties of already approved drugs (metformin, acarbose, rapamycin/analogs, statins, some antihypertensives, etc.). Rapid and affordable identification of new, widely available, and relatively safe anti-aging agents. Immediate repurpose is possible. Refuting unsubstantiated claims of “rejuvenating” properties of some popular drugs.
Assessment of gerotoxicity of drugs and medical interventions
Assessment of the gerotoxicity of chemotherapy, radiation therapy, long-term hormone therapy, immunosuppressants, and general anesthesia. Identification of treatment regimens that minimize accelerated aging as a side effect. Personalization of therapy based on its impact on aging. Objective confirmation of harm, stimulation of the development of less gerotoxic alternatives.
Nutrition and diet optimization
Assessing the geroprotective properties of various diets (Mediterranean, keto, intermittent fasting, calorie restriction, vegan/vegetarianism), the impact of macro- and micronutrients, and eating patterns. Creating scientifically based, personalized nutrition recommendations to slow down aging. Debunking myths and trendy but harmful diets. Preventing age-related diseases at the population level. Identifying diets or eating habits that accelerate aging (e.g., diets high in ultra-processed foods and sugar).
Validation of the effectiveness of nutraceuticals and dietary supplements
Evaluation of vitamins (D, B vitamins, and others), minerals (magnesium, zinc), antioxidants (NAD+ boosters, resveratrol, curcumin), peptides, and other popular “anti-aging” supplements. Scientific proof of the effectiveness of specific substances and their doses for slowing aging. Combating quackery in the dietary supplement market. Providing consumers with reliable information. Exposing ineffective or even potentially harmful supplements, saving consumers money.
Assessing the impact of physical activity and regimen
Evaluating different types of sports and physical activity, their volume, intensity, and frequency of training; the impact of sleep, circadian rhythms (working night shifts), and stress management. Determining optimal personalized training regimens and lifestyle for maximum aging slowdown. Motivating healthy habits. Improving quality of life and functional longevity. Identifying “overtraining” as a factor in accelerating aging and confirming the harmful effects of chronic sleep deprivation and stress.
Environmental factor gerotoxicity assessment
Evaluation of the impact of air, water (heavy metals, pesticides), and soil pollution; noise pollution; and radiation (background radiation, UV). Quantification of health harm in aging terms. Providing a powerful argument for environmental regulation. Stimulating the development of protective measures. Direct evidence of the aging-accelerating effect of pollutants.
Assessing the impact of socioeconomic factors and lifestyle
Research on chronic stress, education level, quality of healthcare, social isolation, and unhealthy habits (smoking and alcohol). To provide evidence for social policy aimed at improving living conditions and reducing health inequalities. Motivating individuals to quit unhealthy habits. Objective confirmation of the detrimental impact of chronic stress, poverty, and social isolation on the rate of aging.
Personalized anti-aging programs
Integrating recommendations on diet, supplements, physical activity, sleep, stress management, and pharmacological geroprotectors. Creating and continuously optimizing a personalized plan to slow down aging for the individual. Real-time monitoring of effectiveness. Maximizing healthy lifespan for the individual. Refuting universal “longevity recipes” and demonstrating the need for personalization.

## Data Availability

The original contributions presented in this study are included in the article. Further inquiries can be directed to the corresponding author.
